# Youngest known case of a pancreatic perivascular epithelioid cell tumor

**DOI:** 10.1002/jpr3.70094

**Published:** 2025-10-09

**Authors:** Laura Gilligan, Wesley C. Judy, Bryan Clary, Timothy Fairbanks, Denise Malicki, Tom K. Lin

**Affiliations:** ^1^ Divisions of Gastroenterology, Hepatology, and Nutrition Rady Children's Hospital San Diego California USA; ^2^ Departments of Pediatrics University of California San Diego La Jolla California USA; ^3^ Surgery University of California San Diego La Jolla California USA; ^4^ Pediatric Surgery Rady Children's Hospital San Diego California USA; ^5^ Pathology Rady Children's Hospital San Diego California USA; ^6^ Pathology University of California San Diego La Jolla California USA

**Keywords:** advanced endoscopy, biliary obstruction, mass, pancreas, pathology

## Abstract

Perivascular epithelioid cell tumors (PEComas) are a rare type of mesenchymal tumor that can arise in any part of the body. As of 2024, 37 cases of pancreatic PEComas had been reported in the literature with patients ranging in age from 17 to 74 years old. This is the youngest reported case of a pancreatic PEComa in an 8‐year‐old female presenting with abdominal pain and test findings of biliary obstruction. Magnetic resonance cholangiopancreatography identified the location, size, and obstructive effects of the mass. These findings prompted the performance of an endoscopic retrograde cholangiopancreatography and endoscopic ultrasound with fine‐needle aspiration to obtain a tissue sample that revealed the diagnosis of PEComa based on the morphological features and immunohistochemistry. Our patient's tumor was benign, successfully resected, and to date, the child has not had tumor recurrence based upon postsurgical serial imaging, which is consistent with most cases of pancreatic PEComas.

## 1 INTRODUCTION

The family of perivascular epithelioid cell tumors (PEComas) includes angiomyolipomas, clear cell “sugar” tumors, and PEComas‐not otherwise specified (PEComa‐NOS). These rare mesenchymal tumors can arise in any part of the body. They are composed of epithelioid cells with clear to eosinophilic granular cytoplasm and most commonly stain positive for melanocytic and myogenic markers. While most PEComas are benign, malignant potential exists. As of 2024, 37 cases of pancreatic PEComa had been reported in the literature with patients ranging in age from 17 to 74 years old^1^, ^2^, ^3^ (Table 1).

**Table 1:** Demographics, treatment, and outcomes in known cases of pancreatic PEComas.

Year	Author	Age	Sex	Location of mass in pancreas	Surgical procedure	Outcome
1996	Zamboni	60	Female	Body	DP	NED
2004	Heywood	74	Female	Head	PPPD	NED
2005	Ramuz	31	Female	Body	SPDP	NED
2008	Perigny	46	Female	Body	Enucleation	NED
2009	Hirabayashi	47	Female	Head	PPPD	NED
2009	Baez	60	Female	Body	DP	NED
2011	Zemet	49	Male	Head	PPPD	NED
2011	Nagata	52	Male	Head	PD	AWD
2011	Xie	58	Female	Head	PD	NED
2012	Finzi	62	Female	Head	Total excision	NED
2012	Singh	38	Female	Tail	Total excision	NED
2013	Al‐Haddad	38	Female	Uncinate process	PD	n/a
2013	Okuwaki	43	Female	Body/tail	DP	NED
2013	Moura	51	Female	Head	PD	AWD
2013	Tummala	n/a	n/a	Head	n/a	n/a
2014	Kim	31	Female	Tail	DP	NED
2015	Petrides	17	Female	Head	PPPD	NED
2016	Wei	58	Female	Body	MP	NED
2016	Mizuuchi	61	Female	Head/body	PD	NED
2016	Collins	54	Female	Head/body	MP	NED
2016	Hartley	31	Female	Tail	DP	n/a
2016	Jiang	50	Female	Head	PD	NED
2017	Zhang	43	Female	Head	PD	NED
2017	Zizzo	68	Male	Head	None	AWD
2018	Sangiorgio	47	Female	Body	n/a	n/a
2018	Sangiorgio	70	Female	Body	n/a	n/a
2018	Hong	35	Female	Head	PD	n/a
2019	Uno	49	Female	Tail	DP	NED
2019	Gondran	17	Male	Head	None (Sirolimus)	AWD
2020	Ulrich	49	Female	Body	DP	n/a
2020	Sinha	59	Female	Tail	DP	n/a
2020	Colon	50	Female	Body	DP	NED
2021	Geng	40	Female	Body	PPPD	NED
2022	Harrison	39	Male	Tail	DP	NED
2022	Sixto	68	Male	Head	PPPD	NED
2024	Tsukita	74	Male	Tail	DP	NED
2024	Yuza	23	Female	Head	PD	NED
2024 (Our case)	Gilligan	8	Female	Head	PD	NED

Abbreviations: AWD, alive with disease; DP, distal pancreatectomy; MP, middle pancreatectomy; n/a, not available; NED, no evidence of disease; PD, pancreaticoduodenectomy; PEComas, perivascular epithelioid cell tumors; mPPPD, pylorus preserving pancreaticoduodenectomy; SPDP, spleen‐preserving distal pancreatectomy.

We present the youngest known case of pancreatic PEComa in an 8‐year‐old female with obstructive jaundice secondary to a solid pancreatic head mass.

## 2 CASE REPORT

An 8‐year‐old female from Guam presented with 6 months of progressively worsening abdominal pain, jaundice, weight loss, and acholic stools. Laboratory studies found a total and direct hyperbilirubinemia of 13 and 10 mg/dL, respectively, alanine aminotransferase (ALT) 421 U/L, and aspartate aminotransferase (AST) 228 U/L. An ultrasound was reportedly completed, though results from Guam were not available. Computed tomography (CT), followed by magnetic resonance cholangiopancreatography showed a noninvasive pancreatic head mass, prompting transfer to the United States.

**Figure 1:**
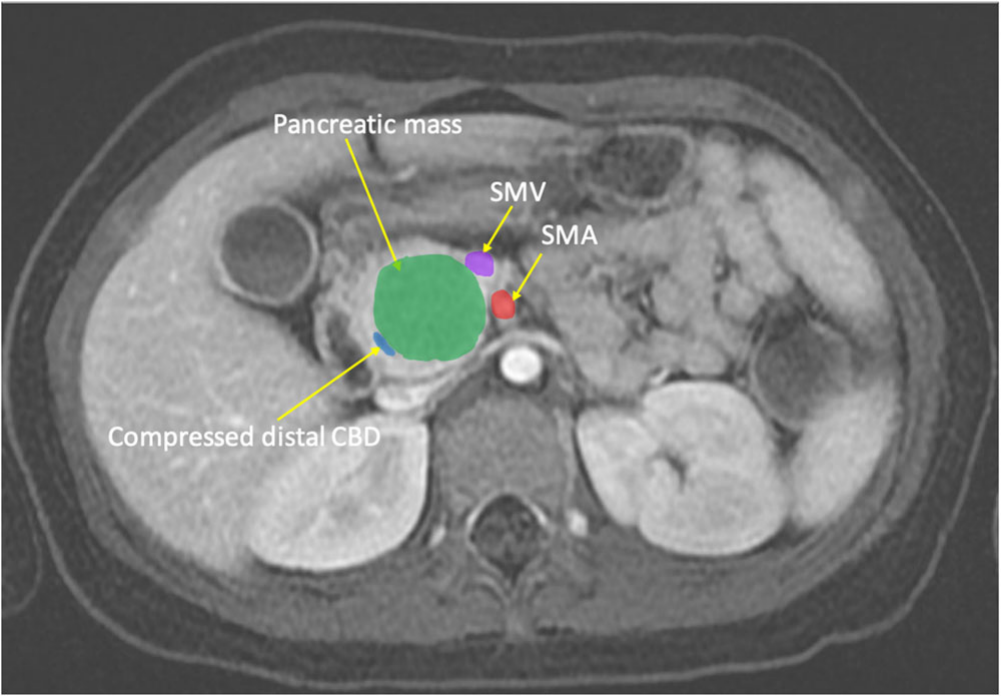
The magnetic resonance cholangiopancreatography image shows a 2.7 × 2.6 × 3.2 cm noninvasive pancreatic head mass compressing the distal common bile duct, mildly displacing the duodenum, abutting the superior mesenteric vein and adjacent to the superior mesenteric artery.

Repeat cross‐sectional imaging (Figure 1) showed a 3 cm solid mass of the pancreatic head partially compressing the distal common bile duct (CBD) and mildly displacing the inferior vena cava, portal vein, duodenum, and distal stomach. There was no evidence of metastasis, nor of abnormal pancreatic duct anatomy such as pancreatic divisum. Partial oncologic workup was pursued and notable for a normal lactate dehydrogenase (187 U/L), normal alpha‐fetoprotein (<2.0 ng/mL), and normal carcinoembryonic antigen (<2.0 ng/mL).

**Figure 2:**
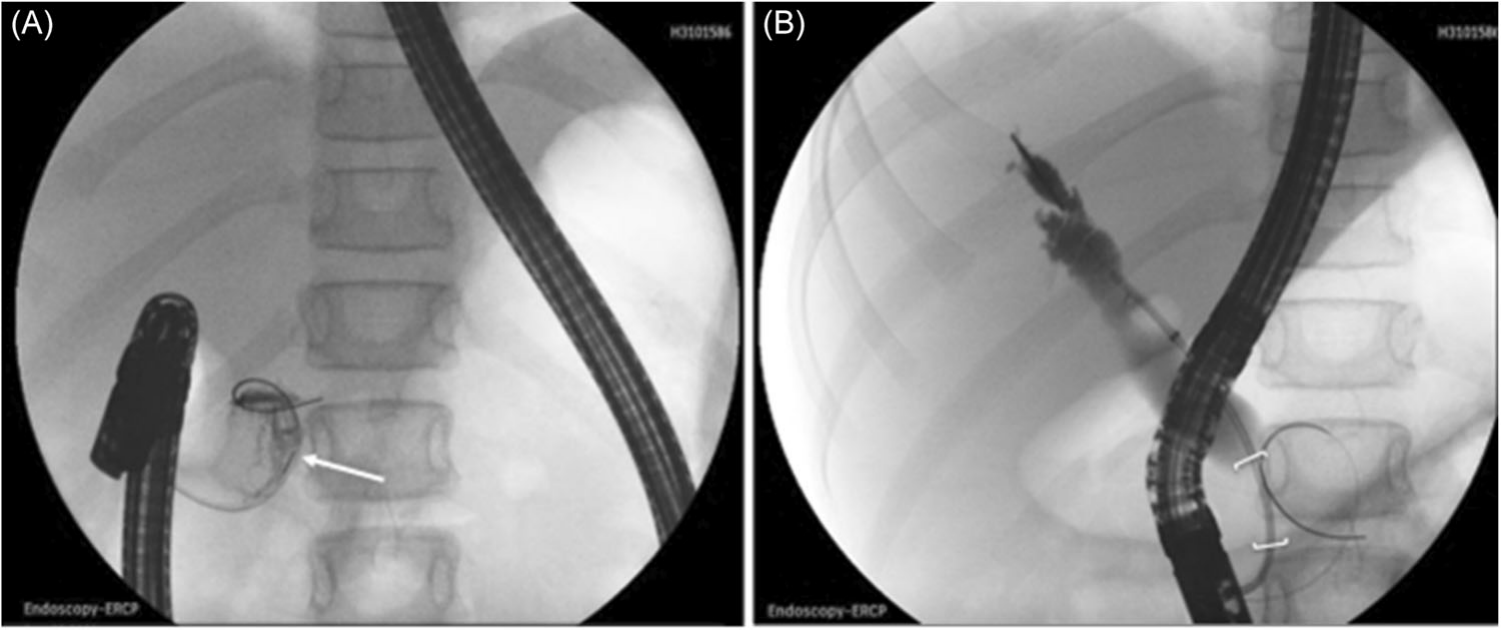
(A) Endoscopic retrograde cholangiopancreatography pancreatogram with contrast opacification of an amorphous intraductal mass (arrow). (B) Cholangiogram with findings of common bile duct stricture (brackets) with upstream main bile duct dilation.

An endoscopic retrograde cholangiopancreatography (ERCP) was performed: pancreatogram of the main pancreatic duct with findings of a large amorphous tissue mass within the head of the pancreas; cholangiogram was notable for a distal CBD narrowing with upstream ductal dilatation extending into the central intrahepatic ducts (Figure 2). A biliary sphincterotomy was performed followed by balloon extraction of a moderate amount of sludge material from the main bile duct. The distal CBD narrowing was brushed and duodenal fluid from the biliary tree and pancreatic duct were aspirated for cytology and tissue pathology. A plastic biliary stent was placed into the CBD. The ventral pancreatic duct showed abnormal filling and did not clearly connect to the main pancreatic duct. Therefore, a pancreatic duct stent was not placed, but a short pancreatic sphincterotomy was performed. The following day, endoscopic ultrasound with fine‐needle aspiration (EUS‐FNA) was performed. Following the procedures, the patient developed epigastric abdominal pain with a lipase of 898 U/L, increased from a lipase of 61 U/L at presentation, consistent with post‐procedural pancreatitis. It was not possible to differentiate a post‐ERCP versus post‐FNA pancreatitis since there was no lipase level between the two procedures.

**Figure 3:**
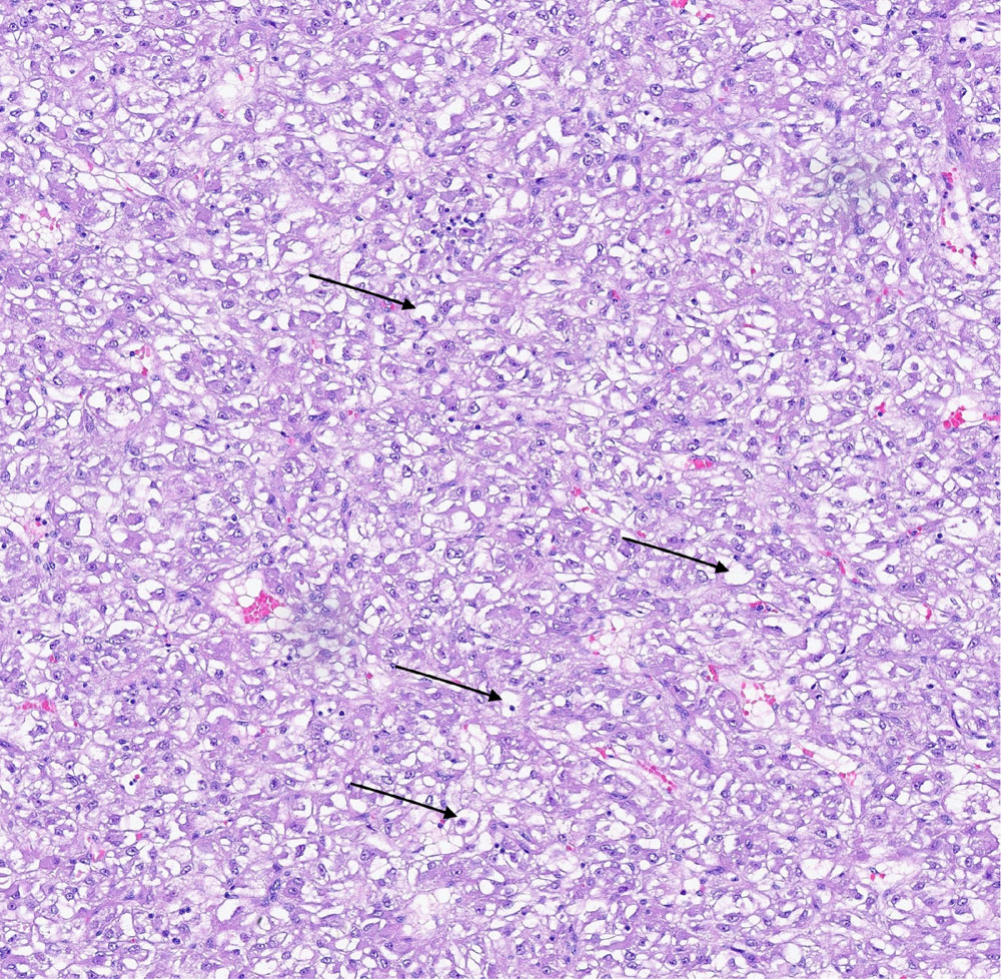
Hematoxylin and Eosin, x20 original objective. Moderately dense proliferation of large epithelioid cells with vacuolated/clear cytoplasm and variably sized nuclei, indicated with arrows.

The cytology brushing of the biliary narrowing and the duodenal aspirates were non‐diagnostic. The FNA specimen revealed loose clusters of large epithelioid cells with vacuolated, eosinophilic cytoplasm (Figure 3) in the background of normal pancreatic acini and ducts. The tumor cells stained positive for HMB45, smooth muscle actin (SMA), inhibin, GATA3, MART1, and TFE3, and negative for CK7, CK20, S100, desmin, and synaptophysin. Collective findings were consistent with the diagnosis of pancreatic PEComa.

The patient underwent pancreaticoduodenectomy with pancreaticojejunostomy, gastrojejunostomy, choledochojejunostomy, and cholecystectomy. The surgical margins were negative and histologically, the tumor was consistent with the FNA specimen, confirmed by immunohistochemistry. There was no evidence of malignant features nor metastases. The patient had an uncomplicated postsurgical course and was discharged on postoperative Day 12. At the 12‐month follow‐up, she was doing well and without evidence of recurrence.

## 3 DISCUSSION

The term “PEComa” was coined by Bonetti et al. in 1992 to describe lesions with epithelioid‐like cells in a perivascular distribution with co‐expression of melanocytic and myogenic markers.^4^ PEComas include angiomyolipomas, lymphangioleiomyomas, clear cell “sugar” tumors, and PEComas‐not otherwise specified. The tumors are characterized by mTOR pathway activating mutations including TSC1 or TSC2 bi‐allelic inactivation, TFE3 gene fusion and FLCN truncating mutations.^5^ PEComas can arise in almost any organ, including the stomach, intestines, liver, pancreas, lung, kidney, and genitourinary organs. Pancreatic PEComas are quite rare with only 37 previously reported cases in the literature.^1^, ^2^, ^3^ Until this case, the youngest reported PEComa patients were 17‐year‐old adolescents^6^, ^7^ (Table 1).

The symptoms of pancreatic PEComas can be vague or absent. Some patients have reported melena,^6^ low‐back pain, or diarrhea.^1^ However, abdominal pain is the most common complaint, present in over half of reported patients.^1^ Our patient had abdominal pain for about 5 months before the appearance of more insidious symptoms. These progressive symptoms prompted further evaluation leading to transfer to the United States for more comprehensive medical care. Aside from PEComa, the differential for a pre‐teen patient with a solid pancreatic head mass includes autoimmune pancreatitis, solid pseudopapillary neoplasm, pancreatoblastoma, insulinoma, adenocarcinoma, lymphoma, and other non‐epithelial tumors.^8^ Given the location of the lesion, it was amenable for EUS‐FNA to obtain the diagnostic specimen for histologic and immunohistochemical evaluation.

Histologically, PEComas arrange as sheets of epithelioid cells with clear to eosinophilic cytoplasm. Distinctive perivascular epithelioid cells are present with the expression of markers for both melanocytes and smooth muscle. Primary tumor features associated with malignant behavior (metastases, local invasion) include size >5 cm, high grade atypia, mitoses >1/50 high power fields, presence of necrosis, and lymphovascular invasion.^9^ Our patient's tumor was 3 cm and met no other criteria, which favored being a benign mass with low likelihood of metastasis. Immunohistochemically, PEComas stain positive for melanocytic (HMB‐45, MiTF, MelanA, and MART1) and myogenic (SMA, desmin) markers. HMB‐45 has been positive in all but one case.^1^, ^2^, ^3^

The optimal treatment of primary pancreatic PEComas involves resection of the mass with negative margins when feasible. Of the 35 cases, including our own, in the existing literature with data on treatment (three cases lack information on treatment), 94 percent underwent surgical resection^1^, ^2^, ^3^ (Table 1). PEComas can reach significant size, yet given the lack of local invasion associated with most primary tumors, resection can still be feasible. This is particularly relevant to PEComas involving the head and uncinate of the pancreas where adjacent visceral vasculature may be displaced but are uncommonly invaded. Systemic medical treatment regimens with modest activity exist including traditional sarcoma agents (anthracycline, gemcitabine) as well as mTOR inhibitors.^7^ Recurrence following resection of pancreatic PEComa in patients without metastases at presentation appears to be quite infrequent, even for those with adverse features.^10^

## 4 CONCLUSION

In summary, pancreatic PEComas are a rare type of mesenchymal tumor. Though typically benign, malignant potential exists and treatment involves resection of the mass. In this report, we describe the presentation, evaluation, and treatment of a pancreatic PEComa in an 8‐year‐old female, the youngest reported patient to be diagnosed with this type of tumor.

## CONFLICT OF INTEREST STATEMENT

The authors declare no conflict of interest.

## ETHICS STATEMENT

We wish to thank the patient and her parents, who provided consent for this case report to be written and discussed.
